# Clinical burden, disease progression, and management strategies in chronic hepatitis B: implications for public health practice

**DOI:** 10.3389/fpubh.2026.1790994

**Published:** 2026-07-09

**Authors:** Shuo Lin

**Affiliations:** Department of Hepatology, Hepatology Research Institute, the First Affiliated Hospital, Fujian Medical University, Fuzhou, China

**Keywords:** antiviral therapy, chronic hepatitis B, clinical burden, disease progression, public health

## Abstract

**Background:**

Chronic hepatitis B (CHB) is one of the most significant global public health issues because it is progressive and has long-term complications, with gaps in real-world management. Longitudinal cohort evidence is required to guide effective disease control strategies.

**Objective:**

This study aimed to evaluate the clinical burden, disease progression, and management strategies in patients with CHB and to identify factors influencing progression and treatment outcomes in a real-world setting.

**Methods:**

The study design was a retrospective longitudinal cohort study conducted at a tertiary-care academic referral centre between January 2020 and December 2025. A total of 520 patients were analysed, all of whom were confirmed to have CHB and followed longitudinally through routine clinical care for a mean duration of 4.1 years. Assessment was made on clinical burden, disease progression and management strategies. Kaplan–Meier analyses were used to determine progression-free survival, and multivariable Cox proportional hazards regression was used to determine independent predictors of disease progression.

**Findings:**

21.5% of patients in the baseline group were found to have significant fibrosis, and 9.8% of the patients had cirrhosis. Follow-up discovered disease evolution in 22.3%, including cirrhosis (11.9%), hepatocellular carcinoma (6.9%), and liver death (5.4%). 46.2% of the patients received antiviral therapy, which was associated with a high rate of progression-free survival. Older age, high levels of HBV DNA, baseline fibrosis, ALT elevation and diabetes mellitus were all independent predictors of disease progression, and antiviral therapy had a very strong protective effect (adjusted HR 0.52, 95% CI 0.36–0.75). It is important to note that almost 20% of eligible patients were not treated, and unstable monitoring was associated with poor clinical outcomes.

**Conclusion:**

CHB is associated with a significant clinical and healthcare burden. Antiviral interventions and regular observation in the early stages of the disease can greatly slow disease progression, and it is therefore crucial to enhance population health interventions to ensure a smooth transition between the guidelines applied and actual practice.

## 1 Introduction

Human HBV (Hepatitis B Virus) belongs to the Hepadnaviridae family. The virus can spread quickly through an infected person’s blood or other bodily fluids. Perinatal, early childhood infection, traditional practices such as tattooing and scarification, sexual contact, blood transfusions, hazardous injection practices, injectable drug use, and occupational exposure of healthcare personnel are common routes of transmission. HBV targets the liver and can result in both acute hepatitis and long-term conditions such as cirrhosis, chronic hepatitis B (CHB) and hepatocellular carcinoma (HCC) (1).

Hepatitis B virus (HBV) infection causes chronic hepatitis B, a liver condition that greatly raises the risk of liver cirrhosis, hepatocellular cancer, and early mortality. Patients with Chronic Hepatitis B also experience psychological, social, professional, and physical effects that may lower their quality of life. Fatigue is one of the physical symptoms, but chronic pain has been shown to impede social interaction and have a detrimental impact on one’s ability to work and care for a family. Depression and social isolation might result from the dread and anxiety brought on by the disease’s growth and worries about its spread to other people (2). 296 million people worldwide suffer from chronic hepatitis B (CHB) infection. Despite excellent vaccines and antiviral medications that potentially prevent cirrhosis and liver cancer by suppressing viral replication, CHB remains the greatest risk factor for primary liver cancer globally. Certain clinical objectives, such as normalisation of liver enzymes (transaminases), Hepatitis B virus (HBV) viral load, and signs of liver cirrhosis, are the main focus of medical care of CHB (3).

Africa has a disproportionate share of the worldwide public health concern posed by hepatitis B virus (HBV) infection. Approximately 4.5 million African children are afflicted, making up 70% of all cases worldwide among children under five. These proportions are concerning for the region. Low awareness, cultural barriers, and insufficient surveillance systems are obstacles that prevent prompt action. Beyond just increasing mortality, HBV also causes serious liver disorders and financial hardships. With only 10% of HBV-positive people receiving a diagnosis, obstacles to eradication include poor testing rates and inadequate treatment coverage. Promising developments include vaccines and antiviral treatments, yet enduring issues such as discrimination, stigma, and low levels of knowledge persist (4).

There is currently insufficient direct evidence on hepatitis B to support official service delivery recommendations. Eight essential strategies are advocated to provide high-quality healthcare services for hepatitis B, based on concepts similar to those used in HIV and HCV treatment. Hepatitis testing, care, and treatment integration with other services (like HIV services and primary care) to increase the efficacy and accessibility of hepatitis services; decentralization of testing and treatment services at primary health facilities to promote access to care supported through task sharing and a differentiated care strategy; community engagement and peer support, strategies to promote and sustain adherence to long-term antiviral therapy, strategies to promote retention in care and track and reengage those disengaged from care. Clinicians, national hepatitis program managers, and other policymakers in health ministries, particularly in low- and middle-income countries who are responsible for creating national hepatitis testing and treatment plans, policies, and guidelines, are the main recipients of these recommendations (5).

The number of persons who are still infected, particularly in less developed countries, will continue to be a public health concern for the foreseeable future, even though the burden of HBV infection has decreased globally as a result of mass immunisation efforts. To reduce the disease’s global burden, preventive policies must be strengthened. HBV vaccination remains one of the best investments in public health today due to its significant benefits and very low cost. Furthermore, enforcing HBsAg testing for blood donations in places where it is not yet mandatory could be a crucial step in preventing infections in clinical settings, as well as in ensuring infection control during invasive procedures and immunising high-risk individuals. Overcoming social and economic obstacles to uphold and enhance prevention policies globally to reduce the disease’s global burden will be a future challenge (6).

Despite chronic hepatitis B being a significant public health issue, there is a lack of evidence about the relationship between clinical disease progression and practical management practices and community health practice. There are gaps in knowledge regarding the impact of screening, access to treatment, and long-term follow-up on outcomes, especially in low-resource settings. This constrains the public health control of CHB by limiting the development of effective, context-specific strategies. The study’s goals were to determine the clinical features and disease progression patterns of people with chronic hepatitis B, assess the effectiveness of current management strategies in this patient population, including monitoring procedures and the use of antiviral therapy, and investigate how these management strategies affect public health-relevant outcomes, specifically the avoidance of complications and the decrease in the overall burden of the disease.

## 2 Methodology

### 2.1 Study design

This study was a retrospective longitudinal cohort study to measure clinical burden, disease course, and treatment plans in patients with chronic hepatitis B (CHB). A tertiary-care health system was used to acquire routinely collected clinical data. A five-year study period from January 2020 to December 2025 was considered to assess real-world disease trends, treatments, and long-term outcomes. This research was approved by the Ethics Committee of the First Affiliated Hospital of Fujian Medical University (Approval No. 2024875).

### 2.2 Study population and sample size

**Figure 1:**
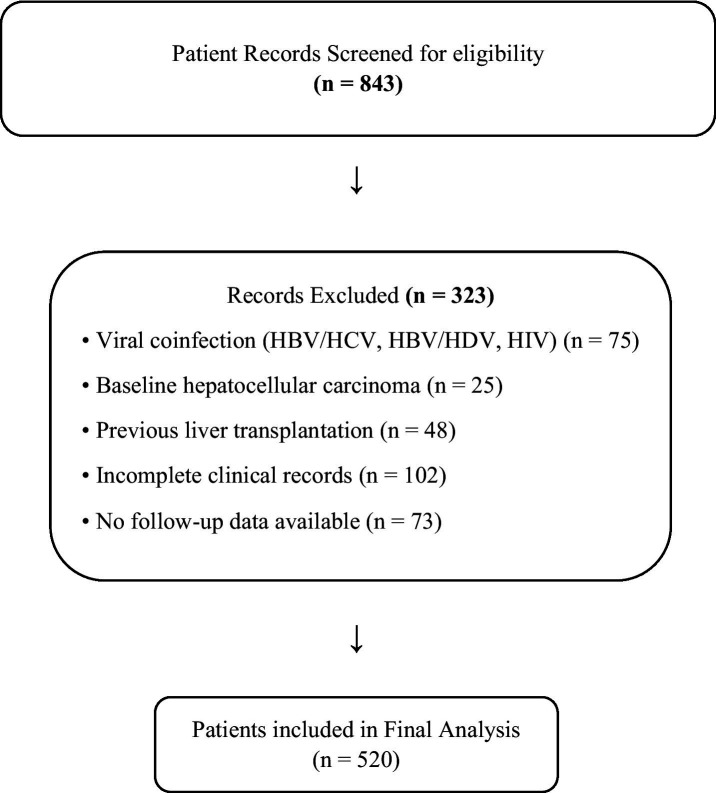
Flow diagram of patient selection.

Using the electronic medical records (EMR) of the First Affiliated Hospital of Fujian Medical University from January 2020 to December 2025, a study population was identified by screening. All patients with chronic hepatitis B who met the inclusion/exclusion criteria were evaluated. Records were examined to ensure that the demographic, clinical, biochemical, virological and follow-up data necessary for analyses were complete. The following patients were excluded because of viral coinfection (hepatitis C virus or hepatitis D virus), previous liver transplantation, hepatocellular carcinoma, incomplete essential medical records, or no follow-up data. A final analysis was performed on 520 adult patients who had been diagnosed with chronic hepatitis B (CHB) after cohort assembly. A patient identification, exclusions and final patient cohort flow diagram is provided in Figure 1. A total of 843 records were screened. After application of eligibility criteria, 75 patients with viral coinfection, 25 with prior liver transplantation, 48 with baseline hepatocellular carcinoma, 102 with incomplete records, and 73 without follow-up information were excluded, resulting in a final cohort of 520 patients.

The participants were managed in accordance with standard institutional clinical practice, and diagnosis and monitoring were based on documented serological and laboratory parameters obtained from electronic medical records. These included hepatitis B surface antigen (HBsAg), HBV DNA quantitation, alanine aminotransferase (ALT), aspartate aminotransferase (AST), and other liver biochemical indices. It was possible to distinguish chronic hepatitis B from chronic hepatitis B by profiles of biochemical and virological activity, in accordance with international standards. The classification of diseases and their eligibility for treatment were based on the recommendations of the World Health Organisation (WHO) (5) and the European Association for the Study of the Liver (EASL) (7). In this retrospective cohort study, all eligible patients who met the predefined inclusion criteria during the study period were included. No formal *a priori* sample size calculation was performed.

#### 2.2.1 Inclusion criteria

Patients were eligible for inclusion if they were aged 18 years or older. They had a confirmed diagnosis of chronic hepatitis B infection, defined as documented persistence of hepatitis B surface antigen (HBsAg) positivity for at least six months. Eligible participants were required to have complete baseline clinical, biochemical, and virological records along with at least one documented follow-up assessment during the study period to enable evaluation of disease progression and treatment outcomes. Patients were categorised as treatment-eligible based on guideline-defined clinical parameters, including serum HBV DNA levels, elevated alanine aminotransferase (ALT) above the upper limit of normal, and evidence of liver fibrosis or cirrhosis. Treatment eligibility generally included HBV DNA levels ≥2,000 IU/mL with persistently elevated ALT levels, or HBV DNA ≥ 20,000 IU/mL regardless of ALT, in selected clinical contexts, as well as patients with significant fibrosis or cirrhosis on imaging or non-invasive assessment. Individuals who met these criteria but did not receive antiviral therapy during the observation period were classified as “eligible but untreated” for analytical purposes.

#### 2.2.2 Exclusion criteria

Patients were excluded from the study if they had evidence of viral coinfections (including hepatitis C virus, hepatitis D virus, or human immunodeficiency virus infection), prior liver transplantation, or hepatocellular carcinoma at baseline evaluation. Additionally, individuals with incomplete or missing medical records, particularly those lacking essential clinical, biochemical, or virological data required for classification and follow-up assessment, were excluded from the analysis.

### 2.3 Data collection and variables

Standardised data abstraction protocols were used to extract demographic, clinical, laboratory and treatment-related information using electronic medical records. The variables gathered were age, sex, comorbidities, biochemical evidence of liver injury, virological parameters and liver fibrosis indicators. Antiviral therapy-related data, such as treatment initiation, duration, and monitoring frequency, were also collected to characterise real-world clinical management between 2020 and 2025.

Other HBV-specific factors, such as quantitative HBsAg levels, HBV genotype, family history of HCC, duration of antiviral therapy, treatment adherence measures, virological breakthrough episodes, HBeAg seroconversion status, and prior history of hepatic decompensation, were not consistently present in the electronic medical record system. They thus could not be reliably included in the analyses.

The routine hepatology assessment included clinical and laboratory assessments by trained physicians. These consisted of a physical examination, liver function tests (ALT, AST), serological testing (HBsAg), and quantitative HBV DNA testing. Hepatocellular carcinoma surveillance was mostly performed using abdominal ultrasound, and cross-sectional imaging (CT or MRI) was performed as indicated by the clinical guidelines. The severity of the disease, treatment status, or patients’ adherence informed the extent of evaluation and follow-up.

Liver fibrosis was evaluated using validated non-invasive indices derived from routinely available laboratory parameters, as not all cases of liver fibrosis were consistently assessed by elastography and histology in this retrospective cohort. The fibrosis-4 (FIB-4) index and the aspartate aminotransferase-to-platelet ratio index (APRI) were used to estimate fibrosis stage, as they are commonly recommended surrogate markers in large-scale and resource-limited research. Significant fibrosis was defined as an FIB-4 score of 2.67 or higher and an APRI of 2.0 or higher; advanced fibrosis or cirrhosis was defined as a higher APRI level, in line with established clinical practice. These indices allowed standardised measurement across all patients, but might not be as diagnostic as elastography or liver biopsy and might introduce misclassification bias, especially at intermediate stages; this consideration was taken into account during data interpretation. Transient elastography (FibroScan), magnetic resonance elastography, and liver biopsy data were available in a subset of patients as part of routine clinical care but were not used as primary criteria for fibrosis classification and were not performed systematically across the cohort. To ensure consistency across all participants, fibrosis staging was performed using non-invasive indices (FIB-4 and APRI) that were uniformly available. Sensitivity analyses using alternative definitions of fibrosis were conducted to assess the robustness of the observations.

Before analysis, data completeness was checked. Patients who lacked the necessary demographic, clinical, biochemical, virological, or follow-up data for outcome assessment were excluded during cohort assembly. In 102 cases (12.1% of those initially screened), the patient’s medical record was incomplete, and a diagnosis was not made. Therefore, a complete-case analysis was conducted for all included individuals.

### 2.4 Clinical burden assessment

The clinical burden was measured by calculating the prevalence of liver-related complications at baseline and follow-up, using such outcomes as severe fibrosis, cirrhosis, and hepatocellular carcinoma. Further measures of burden included liver-related hospitalisations, disease-related complications, and mortality, providing a holistic analysis of the effect of chronic hepatitis B on the healthcare system during the study period.

### 2.5 Disease progression and outcome definitions

Disease progression was defined as the first appearance of cirrhosis, HCC or liver-related death during follow-up. Each progression to significant fibrosis was assessed independently as an intermediate disease outcome and was not a part of the primary composite endpoint for multivariable survival analysis. This was done to minimise the risk of overlap in baseline fibrosis status/outcome.

Hepatocellular carcinoma (HCC) diagnoses were identified from specialist hepatology records and confirmed by radiological findings (contrast-enhanced CT or MRI) and/or histopathological evaluation, where available, in accordance with routine clinical practice. Liver-related mortality was determined from documented clinical records indicating death attributable to liver failure, hepatocellular carcinoma, portal hypertension complications, or other liver-related causes.

Time-to-event was calculated from the date of cohort entry during the 2020–2025 study period to the first occurrence of a relevant clinical endpoint. Progression-free survival was defined as the absence of these outcomes. Follow-up time was measured in years from cohort entry, and patients without disease progression were censored at the date of their last clinical follow-up.

### 2.6 Assessment of management strategies

The evaluation of the management strategies was based on the clinical and virological outcomes of patients receiving antiviral therapy and those not receiving it. The measures used to evaluate the effectiveness of the treatment were viral suppression, biochemical response, and a decrease in disease progression events. The analysis of patterns of initiating, following up and monitoring treatments across the study period identified disjunctions between the recommended care and the actual clinical practice.

Antiviral therapy was evaluated as a time-varying exposure to minimise immortal time bias, with treatment status allowed to change during follow-up. Patients received routine nucleos(t)ide analogues, primarily tenofovir disoproxil fumarate, tenofovir alafenamide, and entecavir. Exposure was defined by therapy initiation during follow-up rather than by baseline classification, and untreated person-time was accumulated until treatment initiation if therapy started later. Treatment effectiveness was assessed using viral suppression, ALT normalisation, and reductions in disease progression events. In contrast, patterns of treatment initiation and monitoring were analysed to identify gaps between guideline-based care and actual practice. Sensitivity analyses confirmed the robustness of associations between antiviral therapy and disease outcomes. Time-varying exposure methods and considerations regarding immortal time bias follow established pharmacoepidemiologic recommendations (8).

### 2.7 Statistical analysis

Baseline characteristics and management patterns were summarised using descriptive statistics. Continuous variables were reported as means with standard deviations or medians with interquartile ranges, and categorical variables as frequencies and percentages. Disease progression over the study period was assessed using Kaplan–Meier survival analysis.

Multivariable Cox proportional hazards regression models were constructed to identify independent predictors of disease progression, with covariates selected based on clinical relevance from prior literature (age, sex, ALT, HBV DNA, fibrosis indices, metabolic comorbidities) and variables showing univariable associations with *p* < 0.10. Highly correlated predictors, particularly ALT, fibrosis indices, and HBV DNA, were evaluated using variance inflation factors (VIF > 5) and not included simultaneously to minimise multicollinearity. The proportional hazards assumption was assessed using Schoenfeld residuals and log-minus-log survival plots. Model performance and goodness-of-fit were evaluated using likelihood ratio tests and Harrell’s concordance index, and sensitivity analyses were conducted using alternative fibrosis measures and by excluding influential observations to assess the robustness of the estimates (9–11). Sensitivity analyses were also conducted with alternative fibrosis definitions: FIB-4 only and APRI only, to ensure the stability of the fibrosis-related associations and estimates of disease progression. There was no material difference across the various definitions of results. Due to the retrospective, observational research design of this study and the limited availability of variables needed to assess the validity of causal inference modelling, propensity score matching and inverse probability weighting analyses were not undertaken. To minimise confounding, however, multivariable adjustment, including major clinical factors associated with disease progression, was performed.

### 2.8 Public health implications

Considering the long-term outlook for providing health services, population health was studied to identify the time lag between diagnosis and initial treatment and the difference between eligibility and actual treatment given. The results were used to identify system-level gaps, and the strategies were intended to reduce disease burden, enhance early intervention, and strengthen chronic hepatitis B control programs.

### 2.9 Follow-up procedures

Follow-ups were performed through routine visits during the study period at the outpatient hepatology clinic. Follow-up schedules were not protocol-based but reflected real clinical practice and guideline-based care. Overall, patients with stable disease were evaluated after 6–12 months, whereas patients with active disease, high viral load, or undergoing antiviral therapy were evaluated more frequently, usually after 3–6 months. Follow-up assessments included repeat laboratory testing (ALT, AST, HBV DNA levels) and evaluation of treatment response and liver disease progression using non-invasive fibrosis indices. Hepatic cell carcinoma surveillance was carried out by means of abdominal ultrasound at a time interval of about 6 months in high-risk individuals (e.g., those with cirrhosis or with advanced fibrosis).

The hepatology specialists handled patient management, and the radiologists and laboratory services assisted with diagnosis. Follow-up frequency and monitoring intensity varied due to differences in patient compliance, clinical condition, and access to healthcare, reflecting real-world practice. The loss to follow-up and inconsistent monitoring were reported and incorporated in the health-system performance evaluation.

Because outcome ascertainment was based on electronic medical records within a closed hospital system, exact loss-to-follow-up rates could not be reliably determined; therefore, patients were censored at their last documented clinical encounter. No patients were included in the cohort if there was no documented follow-up assessment after cohort entry. For survival analyses, participants who did not experience disease progression during the observation period were censored at the most recent clinical follow-up visit.

### 2.10 Ethical considerations

To ensure patient confidentiality, all data were de-identified, and informed consent was not obtained because the study was retrospective. This research was approved by the Ethics Committee of the First Affiliated Hospital of Fujian Medical University (Approval No. 2024875).

## 3 Results

### 3.1 Baseline characteristics of the study population

During the study period, 843 patient records were screened at first glance. After applying a preestablished inclusion and exclusion criteria, patients who were excluded were those in whom there was a viral coinfection (*n* = 75), with liver transplantation (*n* = 25) as their prior treatment, with hepatocellular carcinoma (HCC) as their baseline disease (*n* = 48), with incomplete clinical records (*n* = 102) or without follow-up information (*n* = 73). Of the patients with confirmed CHB, 520 patients were included in the final analysis (Figure 1).

In this study, the cohort, with a mean age of 46.8 ± 12.9 years, was predominantly male (58.5%). At baseline, 55.0% of patients had HBV DNA levels ≥2,000 IU/mL, while the majority (65.8%) were HBeAg-negative. 41.9% of patients had elevated ALT values, indicating persistent hepatic inflammation. At study entry, non-invasive fibrosis assessment showed that 9.8% of patients had advanced fibrosis or cirrhosis (APRI ≥2.0), while 21.5% met criteria for significant fibrosis using the composite FIB-4 ≥ 2.67 or APRI ≥2.0 thresholds. Metabolic comorbidities were common within the cohort, with diabetes mellitus identified in more than one-quarter of patients (27.3%) and overweight or obesity present in 31.7% of participants (Table 1).

**Table 1:** Baseline demographic and clinical characteristics (*n* = 520).

Variable	Overall (*n* = 520)
Age, years (mean ± SD)	46.8 ± 12.9
Male sex, *n* (%)	304 (58.5%)
Female sex, *n* (%)	216 (41.5%)
HBeAg positive, *n* (%)	178 (34.2%)
HBeAg negative, *n* (%)	342 (65.8%)
HBV DNA ≥2,000 IU/mL, *n* (%)	286 (55.0%)
ALT elevated, *n* (%)	218 (41.9%)
AST elevated, *n* (%)	196 (37.7%)
Platelet count <150 × 10^9^/L, *n* (%)	74 (14.2%)
Significant fibrosis (FIB-4/APRI), *n* (%)	112 (21.5%)
Cirrhosis at baseline, *n* (%)	51 (9.8%)
Diabetes mellitus, *n* (%)	142 (27.3%)
Overweight/obesity, *n* (%)	165 (31.7%)

Significant fibrosis defined as FIB-4 ≥ 2.67 or APRI ≥ 2.0, Advanced fibrosis/cirrhosis defined as APRI ≥ 2.0.

### 3.2 Clinical burden of chronic hepatitis B

A substantial clinical burden was observed during the study period (2020–2025). 14.6% of individuals who did not have cirrhosis at baseline developed advanced fibrosis or cirrhosis—6.9% of the cohort as a whole developed hepatocellular carcinoma (HCC). Table 2 showed that 18.1% of patients had liver-related hospitalisations, which is indicative of the morbidity and continued need for healthcare services due to chronic hepatitis B.

**Table 2:** Indicators of clinical burden during follow-up.

Burden indicator	*n* (%)
Any liver-related complication	138 (26.5%)
Liver-related hospitalisation	94 (18.1%)
Progression to cirrhosis	62 (11.9%)
Hepatocellular carcinoma	36 (6.9%)
Liver-related mortality	28 (5.4%)
All-cause mortality	41 (7.9%)

### 3.3 Disease progression patterns

Table 3 indicated that 22.3% of patients had at least one disease progression event during a median follow-up of 4.1 years (interquartile range [IQR] 2.8–5.0 years). Patients with baseline fibrosis, increased ALT, and a high viral load were more likely to progress. Hepatocellular carcinoma developed in 6.9%, and liver-related death occurred in 5.4% of patients. Patients without treatment and those with ongoing virological activity had poorer progression-free survival, according to Kaplan–Meier analysis. Antiviral therapy was analysed as a time-varying exposure, whereby patients contributed follow-up time to the untreated group until therapy initiation and to the treated group thereafter.

**Table 3:** Disease progression events observed during follow-up.

Progression outcome	Number (%)
Progression to significant fibrosis	76 (14.6%)
Progression to cirrhosis	62 (11.9%)
Hepatocellular carcinoma	36 (6.9%)
Liver-related death	28 (5.4%)
Any primary progression event (cirrhosis, HCC, or liver-related death)	116 (22.3%)

Values are presented as the number (percentage) of patients experiencing each outcome during follow-up. Progression to significant fibrosis was analysed separately and was not included in the primary survival endpoint.

Based on approximately 2,132 person-years of observation, incidence rates were 1.69 per 100 person-years for hepatocellular carcinoma, 1.31 per 100 person-years for liver-related mortality, and 5.44 per 100 person-years for the composite disease progression endpoint.

**Figure 2:**
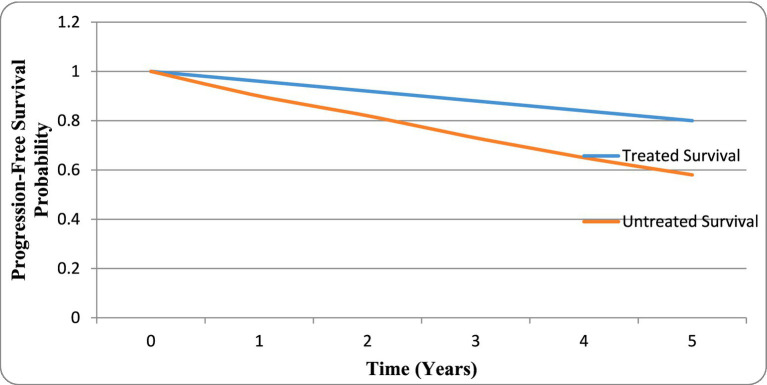
Kaplan–Meier curve for progression-free survival in patients with chronic hepatitis B.

Kaplan–Meier estimates of the progression-free survival in patients with chronic hepatitis B at the 2020–2025 follow-up, by antiviral treatment status. Time-wise, patients who received antiviral therapy had a much higher progression-free survival than untreated patients (Figure 2).

The solid line represents patients on antiviral therapy, whereas the dashed line represents untreated patients.

### 3.4 Predictors of disease progression

Older age, higher HBV DNA, baseline fibrosis, and metabolic comorbidities were independent predictors of disease progression in multivariable Cox proportional hazards analysis. Antiviral treatment showed a strong protective effect, reducing the risk of progression by nearly 50% (Table 4). The multivariable Cox model incorporated time-varying exposure to antiviral treatment to account for treatment initiation during follow-up. The primary composite endpoint for multivariable Cox regression analyses was cirrhosis, hepatocellular carcinoma, or liver-related death. Baseline fibrosis status was included as a predictor variable, as fibrosis progression was not included in the primary endpoint.

**Table 4:** Multivariable predictors of disease progression.

Variable	Adjusted HR	95% CI	*p*-value
Age (per 10-year increase)	1.38	1.18–1.61	<0.001
Male sex	1.21	0.88–1.66	0.24
HBV DNA ≥2,000 IU/mL	1.92	1.31–2.82	0.001
ALT elevation	1.47	1.05–2.05	0.023
Baseline significant fibrosis	2.47	1.68–3.63	<0.001
Diabetes mellitus	1.41	1.02–1.96	0.039
Antiviral therapy	0.52	0.36–0.75	<0.001

**Figure 3:**
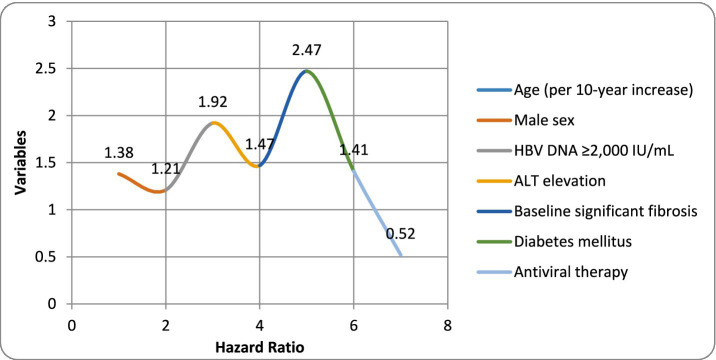
Forest plot of disease progression predictors by seven variables in chronic hepatitis B.

The proportional hazards assumption was not violated for any variable included in the final Cox model, as indicated by Schoenfeld residual testing and visual inspection of log-minus-log survival plots. Figure 3 shows a forest plot of adjusted hazard ratios for factors related to disease progression in patients with chronic hepatitis B from a multivariate Cox proportional hazards regression analysis.

The points are adjusted hazard ratios, where a higher point value means there is a greater chance of disease progression, and vice versa. The variables were age, sex, virological activity, biochemical activity, baseline liver fibrosis, metabolic comorbidity, and antiviral treatment status. Regarding the independent variable, antiviral therapy was observed to reduce the risk of disease progression after adjusting for confounding factors.

#### 3.4.1 Sensitivity analysis of fibrosis definitions

Sensitivity analyses were conducted using different fibrosis definitions, excluding composite scoring, and applying FIB-4 and APRI thresholds separately to evaluate the robustness of the fibrosis classification. The main findings were largely similar across all specifications, with baseline fibrosis consistently showing a strong association with disease progression and hazard ratios consistently in the same direction and magnitude across models.

We used two thresholds for the analysis, FIB-4 ≥ 2.67 and APRI ≥2.0, that were suggested by widely accepted cut-offs for advanced fibrosis in chronic hepatitis B populations and previously validated in real-world cohorts, but recognise that these thresholds may not apply to all clinical settings, which could lead to misclassification, especially in intermediate fibrosis groups. As for fibrosis assessment methods, only a limited number of patients had all three methods (transient elastography-FibroScan, MR elastography, histology) included as part of routine clinical assessment and therefore could not be uniformly included in the main analysis. This is often done in practice, with non-invasive serum-based indices used in large retrospective cohorts.

Further exploratory analyses were performed for each clinical outcome (progression to cirrhosis, hepatocellular carcinoma, and liver-related mortality). There was overall consistency in the direction and strength of associations, with older age, higher HBV DNA levels, baseline fibrosis, and diabetes mellitus being independently associated with greater risk, and antiviral therapy being protective for all endpoints in the composite outcome model. The analysis, however, was constrained by a smaller number of events in each category, which limits its power and thus should be viewed as exploratory.

### 3.5 Management strategies and treatment uptake

Treatment eligibility was assessed retrospectively using predefined clinical criteria applied uniformly across all participants. During the study period, 240 patients (46.2%) received antiviral medication. 19.8% of patients eligible for treatment based on disease activity did not receive it. Only 61.5% of the population received routine surveillance imaging due to variations in monitoring frequency (Table 5).

**Table 5:** Management patterns and treatment uptake.

Management variable	*n* (%)
Antiviral therapy initiated	240 (46.2%)
Eligible but untreated	95 (19.8%)
Regular laboratory monitoring	356 (68.5%)
Regular imaging surveillance	320 (61.5%)
Monitoring gap >12 months	140 (26.9%)

### 3.6 Treatment outcomes

78.3% of treated individuals experienced prolonged viral suppression, while 69.6% experienced a biochemical response. Almost 25% of individuals who received treatment showed signs of fibrosis regression, as shown in Table 6. Hepatocellular carcinoma and disease progression were more common in untreated patients.

**Table 6:** Clinical outcomes by antiviral treatment status.

Outcome	Treated (*n* = 240)	Untreated (*n* = 280)
Viral suppression	188 (78.3%)	54 (19.3%)
ALT normalisation	167 (69.6%)	81 (28.9%)
Fibrosis regression	58 (24.1%)	19 (6.8%)
Any disease progression	34 (14.2%)	82 (29.3%)
Hepatocellular carcinoma	9 (3.8%)	27 (9.6%)

### 3.7 Public health and health-system implications

The results were considerably worse for those who had inconsistent monitoring and a delayed start to treatment. Patients with extended follow-up gaps were more likely to have late-stage disease identification, highlighting systemic shortcomings in continuity of care (Table 7).

**Table 7:** Public health and system-level outcome indicators.

Indicator	Regular monitoring	Irregular monitoring
Disease progression	14.9%	33.6%
HCC detection at an advanced stage	22.2%	51.4%
Liver-related mortality	3.1%	9.8%

## 4 Discussion

These findings provide important real-world evidence from a tertiary-care setting, offering insights into both clinical outcomes and health-system gaps in chronic hepatitis B (CHB) management between 2020 and 2025. The results indicate that CHB remains a significant clinical and public health burden, and over one-fifth of CHB patients develop the disease during a 4.1-year median follow-up. Although there are effective antiviral therapies on the market, major gaps in treatment adherence and monitoring persist, which directly correlate with worse clinical outcomes.

This finding of significant fibrosis in 21.5% and cirrhosis in 9.8% prevalence rates at baseline is in line with the current real-world literature that has found a prevalence of advanced liver disease in 15–30% of CHB patients at diagnosis, especially in tertiary-care settings (7, 9, 10). Follow-up revealed the occurrence of disease progression events (cirrhosis, hepatocellular carcinoma (HCC) or liver-related mortality) in 22.3% of the patients. Rates are similar to those reported in recent cohort studies in Asia, Europe, and North America, which estimate 10–25% progression over 4–6 years in untreated or insufficiently monitored populations (11–13). The overall incidence of disease progression in this cohort is relatively high, but this is due to the cohort being relatively young, with a mean age. The prevalence of cirrhosis in this cohort was only <10% at baseline, and because the cohort of patients seen in a tertiary-care referral centre often has more advanced disease, higher viral activity, and more clinical complexity than would be seen in community-based settings. This referral pattern may have influenced the rates of progress observed and should be taken into account when interpreting the generalizability of the findings.

The high prevalence of HCC (6.9) in this study highlights the continuing cancerogenic risk of chronic HBV infection despite the potency of the nucleoside analogues. Other researchers have also demonstrated that HCC risk is not negligible, especially in patients with a history of baseline fibrosis, cirrhosis, or metabolic comorbidities (14, 15). These results also highlight the necessity of continuous monitoring, as delayed HCC identification was more frequent among patients with inconsistent monitoring (16).

The multivariate Cox regression analysis showed older age, high levels of HBV DNA, fibrosis at the baseline, an increase in ALT and diabetes mellitus as independent predictors of disease progression. These data are closely corroborated by the latest literature, which has shown that chronic viral replication and continuous necroinflammation are the core drivers of fibrosis development and carcinogenesis in CHB (17–19). The correlation between metabolic comorbidities and poor liver outcomes further identifies the escalating interplay between CHB and metabolic dysfunction-related liver disease that has gained more insights over the past years (20–22). Notably, the antiviral treatment was highly protective, reducing the risk of disease progression by approximately 50 per cent. This is in line with large observational studies and meta-analyses that have supported the notion that long-term viral suppression can markedly reduce the risks of cirrhosis, hepatic decompensation and HCC (23, 24). But disease progression in patients under treatment also speaks of the necessity to treat affected individuals as early as possible before the fibrosis gets too severe (25).

Even with explicit guideline recommendations, antiviral therapy was given to only 46.2 per cent of patients in this cohort, and almost 20 per cent of those eligible for treatment were not treated. This is not a novel finding in the rest of the world, and even in high-income nations, where underdiagnosis, disjointed care paths, and restricted access to specialists lead to poor management (1, 2, 26). Various reasons can be attributed to the situation in which a percentage of patients eligible for treatment were not treated with antiviral agents despite clinical evidence. In practice-based clinical settings, the treatment initiation process may be affected by access to healthcare, financial constraints, irregular follow-up attendance, physician bias in risk stratification, and patient-specific issues related to perceived asymptomatic status or long-term therapy. Also, inconsistency in monitoring frequency and timely review of disease activity can contribute to missed treatment opportunities. These results underscore that there are not only clinical treatment gaps but also health-system and behavioural factors that affect adherence and continuity of care.

The lack of uniform laboratory and imaging surveillance also contributed to the unfavourable outcomes, as irregularly followed patients had much greater rates of development of the disease, finding it in an advanced stage, and liver-related death. These results represent a broader universal problem in implementing evidence-based recommendations in clinical practice. Potential strategies to improve long-term monitoring include simplified treatment pathways, improved patient recall systems, and greater integration of HBV care into primary health services.

The results are relevant to hepatitis B control programs. Still, they must be viewed as coming from a single tertiary-care referral centre and may not be representative of community-based or primary-care populations. The World Health Organisation has established ambitious targets for hepatitis elimination by 2030, including expanding diagnostic and treatment coverage and improving long-term retention in care. The treatment and monitoring gaps observed in the present cohort suggest that achieving these goals may remain challenging in some clinical settings (5, 27). Enhancing population-level screening, minimising loss to follow-up, and utilising digital health technologies can have a significant impact on results, especially in resource-limited environments.

The present study was not specifically conducted to assess health-system interventions; however, the gaps in treatment and monitoring are similar across the different studies of the implementation of hepatitis B. HBV education and antiviral access programs, where the community participates in the activities conducted, especially in some countries, have demonstrated better treatment compliance and early detection of the disease. Implementation of these measures can lead to a significant decrease in the development of the disease and mortality due to liver disease in resource-restricted conditions.

### 4.1 Strengths of the study

This study has several significant strengths that contribute to the validity of its results. It is primarily based on a fairly large, well-characterised group of 520 patients with chronic hepatitis B, followed over a very long period of 5 years, which enables an assessment of the true pattern of disease progression and management in the real world. Second, the utilisation of routinely collected electronic medical records reflects real clinical practice, and the information gathered can be translated immediately into health policy and health system planning. The research deliberately collected a broad set of variables, encompassing demographic parameters, biochemical and virological parameters, liver fibrosis indices, treatment initiation, and monitoring practices, thereby enabling the assessment of both clinical and population health burdens in a multidimensional manner. Also, survival analysis and multivariate Cox regression were combined, enabling identification of independent predictors of the disease course while accounting for confounding variables and enhancing the internal validity of the findings. Lastly, by analysing gaps in management, e.g., improper use of antiviral therapy and inconsistent monitoring, the study provides useful information on how to enhance patient outcomes and optimise healthcare resource use. All these methodological strengths contribute to the validity and clinical and translational feasibility of the study’s results.

### 4.2 Limitations of the study

However, several limitations exist in interpreting the results, despite the study’s strengths. The observational nature of the retrospective design also limits the ability to infer causal relationships among treatment, monitoring, and disease outcomes because unmeasured factors may confound observed associations. In addition, the study was conducted within a single tertiary-care health system and may not generalise to other areas or to health systems outside primary-care and rural contexts. The burden of disease, treatment patterns, and disease progression observed at tertiary-care centres may not accurately reflect the situation in the community, as these centres may have more severe cases and greater clinical complexity. Fibrosis assessment was largely based on surrogate biochemical markers (FIB-4 and APRI), with no systematic elastography and/or histological validation. These indices are validated for population-level fibrosis risk stratification. They are widely adopted in real-world cohort studies. Still, some indices may lead to misclassification, especially in patients with intermediate fibrosis stages, potentially underestimating or overestimating fibrosis burden and treatment eligibility. However, the associations remained similar across sensitivity analyses using alternative definitions of fibrosis, providing further confidence in the observed associations.

Event-level data by fibrosis category were not retained in the final consolidated analytical data set, although the baseline fibrosis status was included as an independent predictor in the multivariable Cox regression model. However, analysis of disease progression rates by individual baseline fibrosis stages and estimates of survival based upon individual fibrosis subgroups could not be done. Event data at patient level should be kept for future studies to enable a more detailed fibrosis-stage stratification and risk estimation. Therefore, in MV models, fibrosis was an independent predictor of outcome, but outcome rates within each fibrosis category could not be quantified directly.

Not all information on patient-reported outcomes and socioeconomic factors was available, which may have affected the disease course and the use of health services. Also, selection bias may have been introduced in this study due to the exclusion of patients without data or follow-up information, a common drawback of retrospective cohort study designs. The composite endpoint was utilised for statistical efficiency. Still, individual endpoint analyses may offer further clinical perspective as risk factors may vary among progression to cirrhosis, HCC, and liver-related mortality. During model development, model discrimination and goodness of fit were assessed. Still, detailed performance statistics were not saved in the archived analytical dataset and thus could not be reported after the fact.

Residual confounding cannot be ruled out, although immortal time bias was controlled for by modelling treatment as a time-varying exposure. There could have been other untreated and treated clinical factors that differed. Furthermore, the retrospective data were limited and did not permit the use of more sophisticated causal inference techniques, including propensity score matching or inverse probability weighting. It is important to note that this observed protective effect of antiviral therapy on disease progression should be viewed as associative rather than causal.

In addition, competing-risk analyses were not performed. The combined endpoint included death due to liver-related cause; however, other causes of death could have competed for these patients during the follow-up. Standard Cox proportional hazards models were fitted as detailed information relating to the events was not available at the event level in the archived analytical data set to enable Fine–Gray subdistribution hazard modelling. Therefore, the associations (including the protective effect of antiviral therapy) should be interpreted with due consideration of possible competing-risk bias and should be used with some caution.

Some clinically relevant variables were also not available in the retrospective dataset, including quantitative HBsAg levels, HBV genotype, family history of HCC, treatment adherence, treatment duration, virological breakthrough, HBeAg seroconversion, and prior hepatic decompensation. HBV genotype was not routinely collected in the electronic medical record system and therefore could not be incorporated into the present analyses. The absence of these variables may have limited a more comprehensive assessment of factors associated with disease progression.

### 4.3 Future recommendations

The next research in this field should focus on prospective, multicenter cohort studies, which would improve generalizability and yield stronger causal conclusions. Treatment adherence data, viral genotyping, and lifestyle data will be applied at the patient-level, thus giving a deeper understanding of the development of disease and the response to treatment. In particular, genotype-stratified analyses, particularly in the context of the predominant genotypes of HBV infection in southeastern China (B and C), could enhance risk prediction and be able to identify those at increased risk of disease progression and HCC. Also, health-economic analyses need to be incorporated to determine the cost-effectiveness of the antiviral therapy and surveillance programs. The important public health interventions to reduce disease progression and the risk of hepatocellular carcinoma are strengthening national and regional hepatitis B screening programs, initiating antiviral therapy promptly, and standardising follow-up monitoring. Lastly, the implementation of digital health technologies and telemedicine might help fill gaps in resource monitoring and follow-up, especially in resource-overstrained environments, thereby positively impacting patient outcomes at the population level.

## 5 Conclusion

The paper highlights the high clinical and societal disease burden of chronic hepatitis B. The progression of the disease is common in daily practice, especially observed in untreated and irregularly monitored patients, with the development of advanced fibrosis, cirrhosis, and hepatocellular carcinoma. It has been demonstrated that antiviral therapy can be of great importance in management, as it can considerably decrease the risk of progression. Continuing disparities between treatment eligibility and actual uptake, in conjunction with inconsistent monitoring, point to severe vulnerabilities in existing healthcare delivery systems. It is urgent to address the identified gaps through diagnosis, regular monitoring, and equal access to antiviral treatment to reduce long-term complications and improve population-level outcomes. The results provide practical evidence on public health policies and clinical guidelines to enhance the management of chronic hepatitis B.

## Data Availability

The raw data supporting the conclusions of this article will be made available by the authors, without undue reservation.

## References

[1] Razavi-Shearer D Gamkrelidze I Pan C Jia J Berg T Gray R . Global prevalence, cascade of care, and prophylaxis coverage of hepatitis B in 2022: a modelling study. Lancet Gastroenterol Hepatol. (2023) 8:879–907. doi: 10.1016/S2468-1253(23)00197-8, 37517414

[2] Cohen C Tu T Matthews PC Wang S Hicks J El-Sayed MH . Patient and public health perspectives to inform expansion of hepatitis B treatment guidelines. Lancet Gastroenterol Hepatol. (2025) 10:952–62. doi: 10.1016/S2468-1253(25)00052-4, 40714036 PMC7618342

[3] Ibrahim Y Umstead M Wang S Cohen C. The impact of living with chronic hepatitis B on quality of life: implications for clinical management. J Patient Exp. (2023) 10:23743735231211069. doi: 10.1177/23743735231211069, 38026060 PMC10644750

[4] Faniyi AA Okesanya OJ Manirambona E Oso TA Olaleke NO Nukpezah RN . Advancing public health policies to combat hepatitis B in Africa: challenges, advances, and recommendations for meeting 2030 targets. J Med Surg Public Health. (2024) 2:100058. doi: 10.1016/j.glmedi.2024.100058

[5] World Health Organization. Guidelines for the prevention, diagnosis, care and treatment for people with chronic hepatitis B infection (text extract): executive summary. Infect Dis Immun. (2024) 4:103–5. doi: 10.1097/ID9.0000000000000128, 39391287 PMC11462912

[6] Patel A Dossaji Z Gupta K Roma K Chandler TM Minacapelli CD . The epidemiology, transmission, genotypes, replication, serologic and nucleic acid testing, immunotolerance, and reactivation of hepatitis B virus. Gastro Hep Advances. (2024) 3:139–50. doi: 10.1016/j.gastha.2023.10.008, 39129942 PMC11307719

[7] Lampertico P Agarwal K Berg T Buti M Janssen HL Papatheodoridis G . EASL 2017 clinical practice guidelines on the management of hepatitis B virus infection. J Hepatol. (2017) 67:370–98. doi: 10.1016/j.jhep.2017.03.021, 28427875

[8] Suissa S. Immortal time bias in pharmacoepidemiology. Am J Epidemiol. (2008) 167:492–9. doi: 10.1093/aje/kwm324, 18056625

[9] Terrault NA Lok AS McMahon BJ Chang KM Hwang JP Jonas MM . Update on prevention, diagnosis, and treatment of chronic hepatitis B: AASLD 2018 hepatitis B guidance. Hepatology. (2018) 67:1560–99. doi: 10.1002/hep.29800, 29405329 PMC5975958

[10] Zheng Y Wu J Ding C Xu K Yang S Li L. Disease burden of chronic hepatitis B and complications in China from 2006 to 2050: an individual-based modeling study. Virol J. (2020) 17:132. doi: 10.1186/s12985-020-01393-z, 32859216 PMC7455911

[11] Papatheodoridis GV Chan HLY Hansen BE Janssen HL Lampertico P. Risk of hepatocellular carcinoma in chronic hepatitis B: assessment and modification with current antiviral therapy. J Hepatol. (2015) 62:956–67. doi: 10.1016/j.jhep.2015.01.002, 25595883

[12] Wang X Ren N Liu H Xie J Jie Y Hao C . Global disease and economic burden of noncommunicable diseases attributable to hepatitis B infection: a health economic evaluation study. J Med Virol. (2025) 97:e70519. doi: 10.1002/jmv.70519, 40736217

[13] Adoboi F Mohammadnezhad M. A systematic review study on lived experiences of people living with hepatitis B (PLHB). Glob J Health Sci. (2025) 17. doi: 10.5539/gjhs.v17n1p63

[14] Dagnaw M Muche AA Geremew BM Gezie LD. Prevalence and burden of HBV–HIV co-morbidity: a global systematic review and meta-analysis. Front Public Health. (2025) 13:1565621. doi: 10.3389/fpubh.2025.1565621, 40255371 PMC12006096

[15] Watson AG Mulay AS Gill US. Chronic hepatitis B in 2025: diagnosis, treatment and future directions. Clin Med. (2025) 25:100527. doi: 10.1016/j.clinme.2025.100527, 41192690 PMC12681808

[16] Buti M Riveiro-Barciela M Rodríguez-Frías F Tabernero D Esteban R. Role of biomarkers in guiding cure of viral hepatitis B. Semin Liver Dis. (2020) 40:049–60. doi: 10.1055/s-0039-3401031 31805583

[17] Lv GJ Ji D Yu L Chen HY Chen J He M . Risk of hepatocellular carcinoma occurrence after antiviral therapy for patients with chronic hepatitis C infection: a systematic review and meta-analysis. Hepatol Int. (2024) 18:1459–71. doi: 10.1007/s12072-024-10700-7, 38965190

[18] Huang DQ Tran A Yeh ML Yasuda S Tsai PC Huang CF . Antiviral therapy substantially reduces HCC risk in patients with chronic hepatitis B infection in the indeterminate phase. Hepatology. (2023) 78:1558–68. doi: 10.1097/HEP.0000000000000459, 37184202

[19] Ferreira G Stuurman AL Horsmans Y Cattaert T Verstraeten T Feng Y . Hepatitis B virus infection and the risk of liver disease progression in type 2 diabetic patients with potential nonalcoholic fatty liver disease: a retrospective, observational, cohort study in the United Kingdom clinical practice research datalink. Eur J Gastroenterol Hepatol. (2020) 32:101–9. doi: 10.1097/MEG.0000000000001537, 31651649

[20] Guan MC Ding Q Zhu H. Which risk model can better predict hepatocellular carcinoma in hepatitis B patients with an antiviral treatment duration of over 1 year? J Hepatol. (2024) 80:e160. doi: 10.1016/j.jhep.2023.06.014, 37414258

[21] Inoue T Tanaka Y. Novel biomarkers for the management of chronic hepatitis B. Clin Mol Hepatol. (2020) 26:261–79. doi: 10.3350/cmh.2020.0032, 32536045 PMC7364351

[22] Ertugrul H Ekiz E Islak Mutcali S Tahan V Daglilar E. Chronic hepatitis B: current management and future directions. Diseases. (2025) 13:311. doi: 10.3390/diseases13100311, 41149045 PMC12563165

[23] Yan R Sun M Yang H Du S Sun L Mao Y. 2024 latest report on hepatitis B virus epidemiology in China: current status, changing trajectory, and challenges. Hepatobiliary Surg Nut. (2025) 14:66–77. doi: 10.21037/hbsn-2024-754, 39925891 PMC11806133

[24] Duan L Liu Y Wu D Wen T Huang S Zhu X . Global trends and insights in interferon therapy for chronic hepatitis B: a bibliometric analysis. Virol J. (2025) 22:297. doi: 10.1186/s12985-025-02922-4, 40890755 PMC12400557

[25] Li J Gao Z Bai H Wang W Li Y Lian J . Global, regional, and national total burden related to hepatitis B in children and adolescents from 1990 to 2021. BMC Public Health. (2024) 24:2936. doi: 10.1186/s12889-024-20462-4, 39443929 PMC11515762

[26] Vittal A Ghany MG. WHO guidelines for prevention, care and treatment of individuals infected with HBV: a US perspective. Clin Liver Dis. (2019) 23:417–32. doi: 10.1016/j.cld.2019.04.008, 31266617 PMC9616205

[27] Buti M Bonanni P Ladep N Papatheodoridis G Frühwein M James C . Toward elimination of hepatitis a and B in Europe: vaccination successes, challenges, and opportunities. Expert Rev Vaccines. (2025) 24:373–83. doi: 10.1080/14760584.2025.2502030, 40357587

[28] Cox DR. Regression models and life-tables. J Royal Statis. Soc. (1972) 34:187–202.

[29] Schoenfeld D. Partial residuals for the proportional hazards regression model. Biometrika. (1982) 69:239–41. doi: 10.1093/biomet/69.1.239

[30] Harrell FE. Regression Modeling Strategies: With Applications to Linear Models, Logistic Regression, and Survival Analysis, vol. 608 New York: Springer (2001).

